# Clofazimine Inhibits Human Kv1.3 Potassium Channel by Perturbing Calcium Oscillation in T Lymphocytes

**DOI:** 10.1371/journal.pone.0004009

**Published:** 2008-12-23

**Authors:** Yunzhao R. Ren, Fan Pan, Suhel Parvez, Andrea Fleig, Curtis R. Chong, Jing Xu, Yongjun Dang, Jin Zhang, Hongsi Jiang, Reinhold Penner, Jun O. Liu

**Affiliations:** 1 Department of Pharmacology and Molecular Sciences, The Johns Hopkins University School of Medicine, Baltimore, Maryland, United States of America; 2 Program in Biochemistry, Cellular and Molecular Biology, The Johns Hopkins University School of Medicine, Baltimore, Maryland, United States of America; 3 Center for Biomedical Research at The Queen's Medical Center and John A. Burns School of Medicine at the University of Hawaii, Honolulu, Hawaii, United States of America; 4 Department of Medicine, Feinberg School of Medicine, Northwestern University, Evanston, Illinois, United States of America; 5 Department of Oncology, The Johns Hopkins University School of Medicine, Baltimore, Maryland, United States of America; Oklahoma Medical Research Foundation, United States of America

## Abstract

The Kv1.3 potassium channel plays an essential role in effector memory T cells and has been implicated in several important autoimmune diseases including multiple sclerosis, psoriasis and type 1 diabetes. A number of potent small molecule inhibitors of Kv1.3 channel have been reported, some of which were found to be effective in various animal models of autoimmune diseases. We report herein the identification of clofazimine, a known anti-mycobacterial drug, as a novel inhibitor of human Kv1.3. Clofazimine was initially identified as an inhibitor of intracellular T cell receptor-mediated signaling leading to the transcriptional activation of human interleukin-2 gene in T cells from a screen of the Johns Hopkins Drug Library. A systematic mechanistic deconvolution revealed that clofazimine selectively blocked the Kv1.3 channel activity, perturbing the oscillation frequency of the calcium-release activated calcium channel, which in turn led to the inhibition of the calcineurin-NFAT signaling pathway. These effects of clofazimine provide the first line of experimental evidence in support of a causal relationship between Kv1.3 and calcium oscillation in human T cells. Furthermore, clofazimine was found to be effective in blocking human T cell-mediated skin graft rejection in an animal model *in vivo*. Together, these results suggest that clofazimine is a promising immunomodulatory drug candidate for treating a variety of autoimmune disorders.

## Introduction

Immunosuppressive agents constitute a major class of drugs for the treatment of undesirable or abnormal activation of T lymphocytes and the immune system associated with organ transplantation and autoimmune diseases. Among the most widely used immunosuppressive drugs in the clinic are cyclosporin A (CsA) and FK506, natural products of microbial origin that work through inhibition of intracellular calcium signaling cascade downstream of the T cell receptor (TCR). By recruiting abundant cytosolic immunophilin receptors, each of these immunosuppressive drugs induces the formation of a ternary complex with the calcium, calmodulin-dependent protein phosphatase calcineurin, thereby blocking access to the active site of calcineurin by its substrate, nuclear factor of activated T cells (NFAT), preventing the dephosphorylation and subsequent nuclear translocation of NFAT [1]–[4]. Despite its widespread use among organ transplantation patients [5], CsA and FK506 exhibit significant side effects, particularly nephrotoxicity, which prevents their use for the treatment of a wider range of autoimmune diseases. Since the nephrotoxicity of CsA and FK506 was found to share the same molecular basis as their immunosuppressive effect [6], attention has been turned to other signal transducers downstream of TCR as potential therapeutic targets in recent years.

The Kv1.3 potassium channel [7], [8] has emerged as one of the most promising targets for developing novel immunosuppressants. Although no clear T cell phenotype was observed in Kv1.3 knockout mice [9], several lines of evidence exist in support of a critical role of Kv1.3 channel in the activation and function of human T cells. In particular, Kv1.3 has been shown to play a unique role in effector memory T cell activation [10] and in the pathogenesis of a myriad of important autoimmune diseases. Notably, Kv1.3 has been shown to be highly expressed in auto-reactive effector memory T cells from MS patients [11], [12]. As a result, extensive efforts have been made to discover and develop small molecule inhibitors of Kv1.3 as novel immunosuppressants and immunomodulators. A large number of structurally distinct inhibitors have been found, including UK-78282 [13], WIN17317-3 [14], [15], correolide [16], verapamil [17], and 5-phenylalkoxypsoralens (Psora) [18], among others. Several of the inhibitors have been shown to be effective in animal models of autoimmunity [19]–[21]. However, none has reached the clinic due to lack of potency, specificity, bioavailability or easy access due to structural complexity [22], [23].

The difficulty in finding a clinically useful Kv1.3 inhibitor is not unexpected, as development of a new drug is a tedious and costly process to begin with. To accelerate drug development process, we recently assembled a library of mostly FDA-approved drugs as well as drugs approved abroad and drug candidates that have reached in Phase II clinical trials, known as the Johns Hopkins Drug Library. By systematically screening the library in cell-based assays, we have identified and validated several drugs with novel and previously unknown anti-angiogenic and anti-malarial activities [24]–[27]. Herein, we disclose the identification of clofazimine as a promising hit from another cell-based screen for novel inhibitors of the intracellular TCR signaling pathway leading to the transcriptional activation of IL-2. Through a systematic examination of different steps in intracellular TCR signaling, it was revealed that clofazimine blocked calcium signaling in T cells by directly interfering with the function of Kv1.3 channel. Importantly, it was demonstrated that clofazimine was effective in preventing human T cell-mediated skin graft rejection in a reconstituted mouse model of skin transplantation. As clofazimine has been used as an antibiotic in humans since early 1960s, it has great potential as a novel treatment for many human autoimmune diseases. The distinct structure of clofazimine also offers a novel scaffold for the development of future generations of immunosuppressive and immunomodulatory agents.

## Results

**Figure 1:**
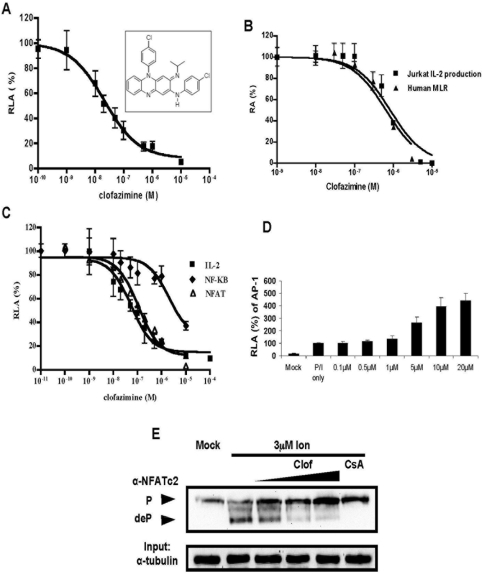
Clofazimine inhibits IL-2 production and NFAT activation in Jurkat T cells. (A) Clofazimine (structure shown in inset) inhibits IL-2 proximal promoter driven-luciferase stimulated by PMA/ionomycin in Jurkat T cells (n = 6). The IC_50_s for reporter assay is 21.8±4.4 nM. (B) Clofazimine inhibits IL-2 production in PMA/thapsigargin-stimulated Jurkat T cells (IC_50_ = 1.10±0.26 µM) and human mixed lymphocyte reaction (MLR) (IC_50_ = 0.90±0.17 µM) (n = 6 each). (C) Clofazimine inhibits NFAT pathway in Jurkat T cells. The IC_50_s of clofazimine in the IL-2, NFAT, NF-κB luciferase reporter assays are 56.0±14.8 nM, 113±30 nM and 2.43±1.25 µM, respectively (n = 6 each). Both reporters were stimulated with PMA/ionomycin. (D) Clofazimine significantly enhances AP-1 luciferase reporter at high concentrations (>1 µM, n = 6). The reporter was also activated by PMA/ionomycin. (E) Clofazimine inhibits dephosphorylation of endogenous NFATc2 in response to ionomycin treatment. NFATc2 and α-tubulin were detected by Western blot using specific antibodies.

The signaling pathway emanating from TCR and leading to the transcriptional activation of the IL-2 promoter is dependent on the second messenger calcium and the calcium and calmodulin-dependent protein phosphatase calcineurin that has been shown to be a common target for both cyclosporine A and FK506 [2]–[4]. We thus engineered a reporter T cell line by stably integrating a luciferase reporter gene under the control of the minimal human IL-2 proximal promoter into the genome of Jurkat T cells. Upon stimulation with PMA and ionomycin, a 20-fold increase in luciferase activity was observed (data not shown). Using the reporter T cell line, we screened the Johns Hopkins Drug Library at a final concentration of 10 µM for each drug using the IL-2 reporter assay in 96-well format [24]–[26]. The known immunosuppressive drugs CsA and FK506 were both positive hits, validating the screen. One of the most potent novel hits was identified as clofazimine (Fig. 1), a known anti-mycobacterial drug that has been used for the treatment of leprosy [28], [29].

The ability of clofazimine to inhibit TCR-mediated IL-2 production was confirmed using transiently transfected IL-2 luciferase reporter gene in Jurkat T cells and a separately prepared clofazimine stock solution. Clofazimine inhibited PMA/ionomycin-stimulated IL-2 luciferase reporter gene activation with an IC_50_ of 22 nM (Fig. 1A). It also inhibited the activation of endogenous IL-2 promoter in response to PMA and thapsigargin with an IC_50_ of 1.1 µM (Fig. 1B). Importantly, clofazimine inhibited human mixed lymphocyte reaction with an IC_50_ of 0.9 µM (Fig. 1B), similar to its effect on endogenous IL-2 production in Jurkat T cells.

The transcription activation of the IL-2 promoter is dependent on three key transcription factors, NF-AT, NF-κB and AP-1. We thus determined whether clofazimine affected the activation of each of those transcription factors using their respective luciferase reporters. As shown in Fig. 1C, while the NFAT luciferase reporter was as sensitive to clofazimine as the IL-2 promoter-driven luciferase reporter gene, the NF-κB luciferase reporter is about 40-fold less sensitive to clofazimine. In contrast, the AP-1 luciferase reporter gene activity was enhanced, rather than inhibited, by higher concentrations of clofazimine (Fig. 1D). A similar stimulation of the AP-1 reporter was also observed at higher concentrations of CsA (Fig. S1A), suggesting that clofazimine may affect the same signaling pathway as CsA.

The selective inhibitory effects of clofazimine on both NFAT and NF-κB over AP-1 suggested that it is likely to affect the activation of their common upstream regulator, calcineurin [1], [3], [4]. To assess this possibility, we determined whether clofazimine, like CsA and FK506, affected the dephosphorylation of endogenous NFAT in response to ionomycin treatment. Similar to CsA, clofazimine inhibited ionomycin-induced dephosphorylation of NFATc2 in a dose-dependent manner (Fig. 1E). In addition, clofazimine also blocked the ionomycin-induced nuclear translocation of NFAT in Jurkat T cells (Fig. S1B–C). Together, these results indicated that clofazimine inhibited the activation of calcineurin *in vivo*. We next examined the effects of clofazimine on the activity of calcineurin *in vitro*. Clofazimine had no effect on the enzymatic activity of calcineurin with either *para*-nitrophenylphosphate or immunoprecipitated endogenous NFATc2 as a substrate (Fig. S2A–B). Nor did it affect the binding of GST-NFATc2 to recombinant calcineurin (Fig. S2C). Interestingly, when the association between the N-terminal fragment of NFAT and the constitutively active form of calcineurin (CnΔC) was examined in a mammalian two-hybrid assay, clofazimine inhibited the calcium-dependent NFAT-calcineurin interaction in a dose-dependent manner (Fig. S2D). Thus, clofazimine appeared to act at a step upstream of calcineurin activation *in vivo*, raising the possibility that it affected either the release of intracellular calcium or calcium influx through the plasma membrane calcium channels.

**Figure 2:**
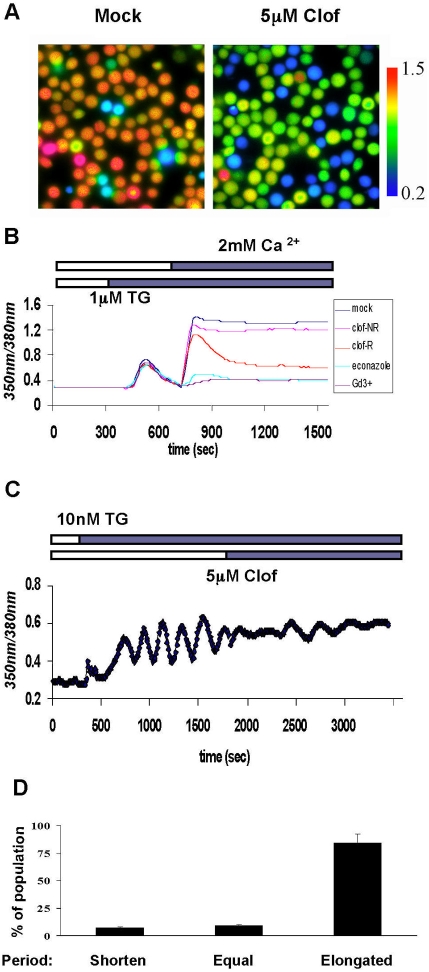
Clofazimine interferes with calcium influx in Jurkat T cells. (A) Calcium influx was inhibited by clofazimine in a heterogeneous fashion. Clofazimine was added 5 minutes before stimulation with 1 µM TG. Representative images were taken 30 minutes after 2 mM calcium was added. The color gradient represents fura-2 350 nm/380 nm excitation ratio. (B) Effects of clofazimine on store-depletion induced calcium influx. Typically the cells can be divided into 2 groups, the responsive (red) and none-responsive (pink) populations. Each curve represents average signal of 20 cells from the same field in (A) (The results were reproduced 10 times under the same condition). (C) Effect of clofazimine on calcium oscillation in Jurkat T cells (representative of 56 cells). The oscillation was stimulated by 10 nM TG. (D) Clofazimine elongated oscillation period in more than 80% of Jurkat T cells (Results represented 3 experiments, at least 80 cells were counted for each experiment).

We employed live cell imaging to determine the effect of clofazimine on changes in intracellular calcium concentrations in response to thapsigargin treatment. Using the calcium indicator dye Fura-2AM, we were able to observe entry of calcium into Jurkat T cells upon treatment of cells with thapsigargin followed by addition of 2 mM Ca^2+^ into the extracellular medium (Fig. 2A). Pretreatment of Jurkat T cells with known CRAC channel inhibitors econazole or gadolinium abrogated calcium entry as expected (Fig. 2B). We noticed that Jurkat T cells exhibited heterogeneity in their response to clofazimine with varying degrees of inhibition at a given concentration of clofazimine. For example, when cells were preincubated with clofazimine for 5 min, the calcium entry of only about a quarter of cells are inhibited to near completion by the drug while that in remaining cells was blocked to a lesser degree (Fig. 2B). When the preincubation time was increased to up to 2 h, there was a time-dependent increase in the proportion of cells that became sensitive to clofazimine (Fig. S3A–B). The inhibitory effect of clofazimine on the extracellular calcium influx suggested that it might affect the CRAC channel. We thus examined the effects of clofazimine in reconstituted CRAC channels using ectopically expressed CRACM1 (Orai1), CRACM2 or CRACM3 subunits co-expressed with STIM1 in HEK 293T cells. But we observed no effects of clofazimine on the reconstituted CRAC current (Fig. S3C), ruling out the possibility that clofazimine directly interacts and interferes with the known components of the CRAC channel.

Given that clofazimine inhibited calcineurin activation in T cells, we next determined whether clofazimine affected the oscillation frequency Ca^2+^ entry in Jurkat T cells, which has been shown to be critical and selective for sustained activation of calcineurin and NFAT to drive cytokine gene expression [30]. Indeed, addition of clofazimine significantly disrupted the oscillation patterns of the store-operated Ca^2+^ entry induced by a low concentration of thapsigargin [31]. It both decreased the amplitude and increased the period of the calcium oscillation (Fig. 2C). In contrast to the partial response of calcium influx to clofazimine detected by Fura-2AM (Fig. 2A–B), over 80% of cells exhibited elongation of the oscillation period upon treatment with clofazimine, indicating that this effect is statistically significant (Fig. 2D).

**Figure 3:**
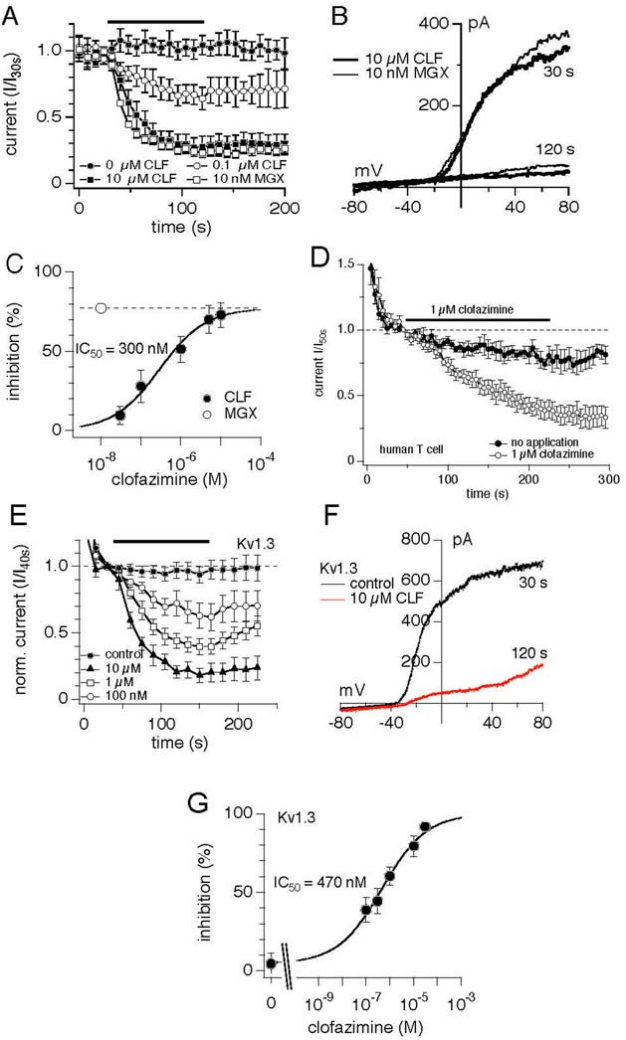
Clofazimine inhibits Kv1.3 channel. (A) Averaged time course of Kv1.3 currents measured in Jurkat T cells in the absence and the presence of clofazimine (CLF) or margatoxin (MGX). Various concentrations of CLF (n = 5 each) or 10 nM MGX (open squares, n = 5) were applied as indicated by the black bar. Currents were elicited by applying a ramp protocol from −100 mV to +100 mV over a span of 50 ms and acquired every 2 s. Holding potential was −80 mV. Current amplitudes were extracted at +80 mV and plotted versus the time of the experiment. Currents were normalized to cell size and plotted as pA/pF. Error bars indicate S.E.M. (B) Current-voltage relationship of Kv1.3 currents taken from example cells and extracted before (30 s) or after application (120 s) of either 10 µM CLF (thick trace) or 10 nM MGX (thin trace). (C) Dose-response curve of the inhibitory effect of CLF (closed circles, n = 5 each). Data were normalized to the current measured before application at 30 s as I/I_30s_. The inhibition was measured at the end of application at 120 s. A dose-response fit to the data resulted in an IC_50_ of 300 nM and a Hill coefficient of 0.75. The maximum inhibitory effect of CLF was compared to the inhibitory effect of 10 nM MGX (open circle, n = 5), a saturating concentration to assure Kv1.3 inhibition. The dashed line indicates the maximum inhibitory effect of MGX. (D) Clofazimine inhibits Kv1.3 in primary human T cells. Average development of Kv1.3 currents measured in human T cells (protocol approval number RIRC QMC RA-2004-048) in control cells (closed circles, n = 5) and with superfusion of external standard solution supplemented with 1 µM clofazimine (open circles, n = 5) as indicated by the black bar. Error bars indicate S.E.M. Data were normalized to the time of application, averaged and plotted versus time of the experiment. Holding potential was −80 mV. Voltage ramp protocol and solutions are described in methods. (E) Current behavior of heterologous mouse Kv1.3 expressed in L929 cells plotted over time in response to increasing concentrations of clofazimine, applied through a wide-mouth glass pipette at the time indicated by the black bar (control, closed circles, no application, n = 7; 10 µM, closed triangles, n = 4; 1 µM, open square, n = 6; 100 nM, open circles, n = 5). Currents were measured by application of a ramp protocol from −100 mV to +100 mV over 500 ms and given at 5 s intervals. Current amplitudes were assessed at +80 mV, normalized to the current amplitude at 40 s, averaged and plotted versus time. Error bars indicate S.E.M. (F) I/V curve of a representative cell expressing mouse Kv1.3 with control I/V (black) extracted at 40 s after whole-cell establishment and the I/V for 10 µM clofazimine extracted at the end of application (red, 160 s). (G) Concentration-response behavior of mouse Kv1.3 expressed in L929 cells to increasing concentrations of clofazimine (n = 4–7). A fit to the data gave an IC_50_ of 470 nM with a Hill coefficient of 0.5.

The pronounced effects of clofazimine on the oscillation patterns of calcium entry, together with the lack of effect of clofazimine on reconstituted CRAC current, raised the possibility that it may affect other channels, particularly potassium channels, which are known to regulate the driving force for Ca^2+^ through open CRAC channels. We thus determined the effects of clofazimine on the activity of various known channels expressed in activated T cells. As shown in Fig. 3A and B, clofazimine had a dramatic effect on Kv1.3 current in a time- and dose-dependent manner. It inhibited the Kv1.3 potassium current with an IC_50_ of 300 nM and a Hill coefficient of 0.75 (Fig. 3C), consistent with its potency for the inhibition of both endogenous IL-2 production in Jurkat T cells and the human mixed lymphocyte reaction (Fig. 1B). In addition to Jurkat T cells, we also determined the effect of clofazimine on Kv1.3 activity in primary human T cells and a similar inhibitory effect was observed as in Jurkat T cells (Fig. 3D). In contrast to Kv1.3, the activity of Ca^2+^-activated potassium channels (IKCa1) [32] and non-selective cation channels (TRPM4) [33], remained unaffected by clofazimine at up to 10 µM concentration when cells were perfused with intracellular solutions in which Ca^2+^ was buffered to 1 µM (data not shown).

To further investigate the specificity of clofazimine, we conducted a series of experiments testing the drug against several heterologously expressed potassium channels, including mouse Kv1.3 [34]. As shown in Fig 3E and 3F, clofazimine strongly suppressed mouse Kv1.3 stably expressed in L929 cells with an IC_50_ of 470 nM and a Hill coefficient of 0.5 (Fig. 3G). All other Kv channel species tested (Mouse Kv1.1, rat Kv1.2, human Kv1.5 and mouse Kv3.1 [34] proved considerably less sensitive to clofazimine, blocking less than 50% of current at a 10 µM concentration (Fig. S4). This indicates that their IC_50_ values for clofazimine are above 10 µM. Interestingly, there seems to be some voltage dependence to the effect on Kv channels other than Kv1.3, since the clofazimine block is smaller at 0 mV compared to +80 mV (Fig. S5). Since most cells do not depolarize beyond 0 mV, at least not for appreciable amounts of time, this represents the more physiologically relevant parameter in regards to the inhibitory effect of clofazimine. Taken together, these data suggest that clofazimine is highly selective for the Kv1.3 channel.

**Figure 4:**
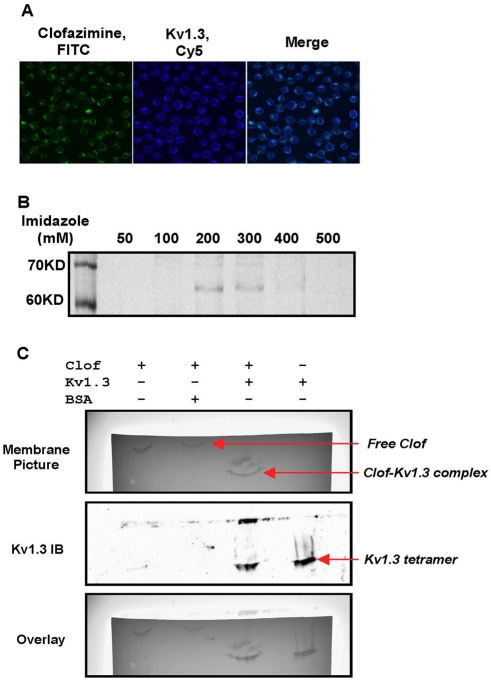
Interaction between clofazimine and Kv1.3 *in vivo* and *in vtro.* (A) Clofazimine colocalizes with Kv1.3 on the plasma membrane of Jurkat T cells. Clofazimine was visualized in FITC channel while Kv1.3 was stained by Cy5 secondary antibody. (B) SDS-PAGE analysis of His-Kv1.3 purified from 293 T cells upon staining with Coomassie blue. Recombinant Kv1.3 protein was found in the 200 and 300 mM imidazole elution fractions. (C) Gel shift of clofazimine by Kv1.3 on native polyacrylamide gel. 10 µM Clofazimine was incubated with buffer alone (lane 1), 2 µM BSA (lane 2) and 2 µM Kv1.3 (lane 3) for 30 minutes before the samples were resolved on a native polyacrylamide gel. Proteins and clofazimine were transferred to nitrocellulose membrane. The presence of clofazimine was apparent by its intense organge/yellow color (member picture) and Kv1.3 was detected by Western blot analysis. The membrane image was taken in the same field after HRP detection was completed.

Kv1.3 is known to exhibit a polarized cell surface expression pattern, which can be visualized using polyclonal antibodies in conjunction with Cy5-labeled secondary antibodies [35] (Fig. 4A). We took advantage of the intrinsic fluorescence of clofazimine, which can be detected using the same filter as for FITC and compared the distribution patterns of clofazimine and Kv1.3. Indeed, clofazimine displayed the same polarized subcellular localization pattern as that of Kv1.3 (Fig. 4A), suggesting that clofazimine is likely to be associated with Kv1.3 *in vivo*.

Next, we assessed the direct interaction between clofazimine and purified recombinant Kv1.3 *in vitro*. His-Kv1.3 was expressed in 293T cells and purified to near homogeneity (Fig. 4B) as described previously [36]. The interaction between purified Kv1.3 protein and clofazimine was assessed by taking advantage of the difference in their mobility in native polyacrylamide gels. Free clofazimine migrated quite slowly in the gel (Fig. 4C, top panel). Addition of BSA did not affect the gel mobility of clofazimine. Upon mixing with recombinant Kv1.3, however, clofazimine co-migrated with Kv1.3, as judged by the overlap of the Kv1.3 protein band revealed by Western blot and the colored clofazimine band (Fig. 4C, bottom panel). The shift in gel mobility of clofazimine by recombinant Kv1.3 strongly suggests that clofazimine forms a complex with recombinant Kv1.3 by directly binding to the protein.

**Figure 5:**
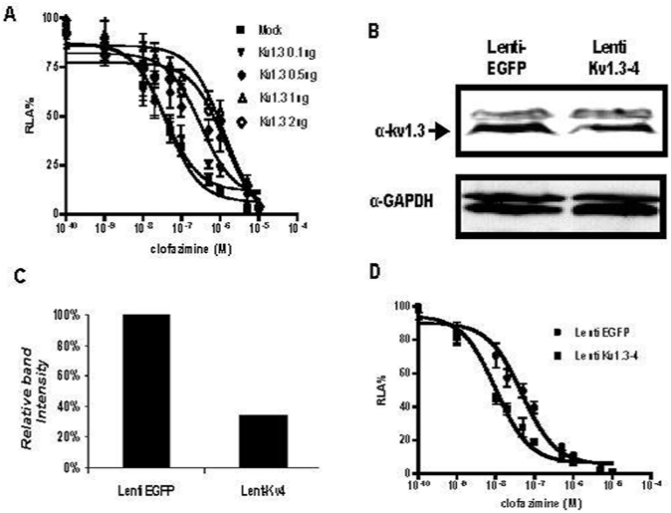
(A) Over-expression of Kv1.3 in Jurkat T cells resulted in resistance of the IL-2 luciferase reporter to CLF. The IC_50_s for different curves (Kv1.3 overexpression from low to high) are 41.6±8.4 nM, 41.0±14.5 nM, 386±214 nM, 1.37±0.52 µM and 1.46±0.63 µM (n = 6). (B) Kv1.3 knockdown resulted in an increase in sensitivity of the IL-2 luciferase reporter to CLF. The IC_50_s for control EGFP-siRNA lentiviral transduced Jurkat T cells (control) was 49.9±13.0 nM, while that for shKv1.3-4 transduced cells was 10.5±2.4 nM (n = 6). (C) Lentivirus-mediated knockdown of Kv1.3 assessed by Western blot. (D) Quantitation of Kv1.3 knockdown efficiency. After normalization against GAPDH, lenti-Kv1.3-4 decreased Kv1.3 protein level by 70% in comparison to the control (Lenti-EGFP).

To further assess the physiological relevance of Kv1.3 as a molecular target for clofazimine, we first determined the effect of ectopic overexpression of Kv1.3 on the sensitivity of the IL-2 reporter gene to clofazimine. As shown in Fig. 5A, overexpression of Kv1.3 led to a gain in resistance of the IL-2 reporter to clofazimine in a dose-dependent manner. At the highest concentration of Kv1.3 expression plasmid used (2 µg), there was a 35-fold increase in the IC_50_ value of clofazimine. Next, we downregulated the expression of endogenous Kv1.3 using lentivirus-mediated RNA interference. Of a total of nine RNAi constructs tested, the most effective construct, shKv1.3-4, partially downregulated the protein level of Kv1.3 by ca. 70 % (Fig. 5B, C). A comparison of the dose-response curves of Jurkat cells transduced with shKv1.3-4 lentiviruses and those transduced with viruses carrying a control shRNA against EGFP revealed that knockdown of Kv1.3 increased the sensitivity of the IL-2 luciferase reporter to clofazimine with a nearly 5-fold decrease in the IC_50_ values (Fig. 5D). In contrast, knockdown of Kv1.3 had no effect on the sensitivity of the IL-2 luciferase reporter to CsA (Fig. S6). The changes in the sensitivity of the IL-2 reporter gene to clofazimine upon overexpression or knockdown of Kv1.3 provide strong support for the notion that Kv1.3 is a specific molecular target of clofazimine.

**Figure 6:**
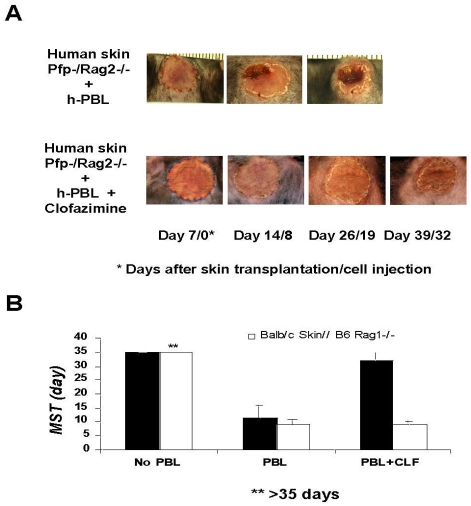
Clofazimine inhibits human T cell-mediated skin graft rejection in immunodeficient mice. (A) Representative skin grafts at different days post-transplantation. Human foreskin was transplanted onto Pfp/Rag2−/− mice. 1.0×10^8^ human peripheral blood lymphocytes (PBL) were adoptively transferred into each animal at Day 7 post-transplantation. Administration of clofazimine or carrier control (olive oil) was also initiated at Day 7. * Days after skin transplantation/cell transfer. (B) Effect of clofazimine on the mean survival time of transplanted human (n = 5) and mouse (n = 4) skin grafts. The mouse skin transplantation was performed using Balb/c mice as skin donors, B6 Rag1−/− mice as recipients and PBL from B6 for adoptive transfer.

Kv1.3 has been implicated in T cell activation and has served as a molecular target for developing novel immunosuppressive agents [22], [23]. Given that clofazimine is already used in the clinic, albeit for a completely different indication, we wondered whether it is efficacious in animal models of organ transplantation. Initial experiments using mouse skin or heart transplant models revealed no beneficial effects of clofazimine in those models. These negative results are not surprising given that Kv1.3 plays distinct roles in humans and rodents, as it has been shown that Kv1.3 is dispensable in mice due to the up-regulation of other chloride channels [9]. Consistent with this notion, we also failed to observe a dose-dependent inhibition of IL-2 production in primary mouse T cells (Fig. S7A) and murine mixed lymphocyte reaction (Fig. S7B). Moreover, similar results were obtained for mixed lymphocyte reaction using cells derived from rats, making it difficult to evaluate the *in vivo* effects of clofazimine using well-established animal models. To overcome this problem, we turned to a model of reconstituted human T cell-mediated human skin rejection in immunodeficient mice [37]. We thus transplanted human foreskin into Pfb-Rag2−/− mice that lack T, B and NK cells. Upon healing of the skin graft for about 7 days, a total of 100 million human peripheral blood lymphocytes from an unrelated donor were adoptively transferred into the same animals. The animals were administered orally either olive oil (control) or clofazimine at 50 mg/kg/day for a total of 10 days (Fig. 6A). For the control group, the transplanted foreskin was rejected with a median survival time of 11 days (Fig. 6B). For the group treated with clofazimine, the skin survived even beyond the cessation of the drug treatment with a mean survival time of 35 days (Fig. 6B), which is comparable to the efficacy for FK506 treatment (data not shown). It is noteworthy that in a parallel experiment using murine skin and total murine T cells, clofazimine had no effect on the survival of murine skin transplant (Fig. 6B). Together, these results demonstrated that clofazimine is uniquely effective in inhibiting human T cell-mediated graft rejection with no significant effect on murine T cells.

## Discussion

The intracellular TCR-mediated signal transduction pathway leading to IL-2 transcription is essential for the activation of quiescent T cells and as such has served as a reliable model system to discover and evaluate new immunosuppressive agents. In addition to the discovery of FK506 using this model system [38], other immunosuppressive agents have been discovered from different chemical libraries [39], [40]. By screening a library of known drugs (HDL), we identified clofazimine as a novel inhibitor of this signaling pathway. Further mechanistic deconvolution by systematically examining the known steps in this signaling pathway led to the identification of Kv1.3 as a physiologically relevant target for clofazimine. The selective inhibition of Kv1.3 by clofazimine accounts for the perturbation of calcium oscillation patterns by the drug and the distinct effects of clofazimine on the intrinsic enzymatic activity of calcineurin *in vitro* and the calcineurin-mediated NFAT dephosphorylation *in vivo*.

Several lines of evidence were obtained that support Kv1.3 as a major molecular target for clofazimine. In addition to the inhibition of channel activity of ectopically expressed Kv1.3, it was found that clofazimine demonstrated remarkable selectivity for Kv1.3 over other related Kv channels including Kv1.1, Kv1.2, Kv1.5 and Kv3.1 (Fig. S4, S5). More importantly, we not only observed colocalization of clofazimine with Kv1.3 in live cells, but also denmonstrated that clofazimine could directly associate with purified recombinant Kv1.3 protein in native polyacrylamide gel as judged by the co-migration of the otherwise less mobile clofazimine and faster migrating Kv1.3 protein (Fig. 4C). To further assess the physiological relevance of Kv1.3 as a target of clofazimine, we also determined the activities of a well-known Kv1.3 inhibitor, Psora-4, in several assays (Fig. S8). Gratifyingly, Psora-4 displayed an activity profile quite similar to clofazimine, including heterologous inhibition of calcium influx in Jurkat T cells (Fig. S8A–B vs. Fig. 2A), selective inhibition of IL-2 and NFAT luciferase reporter genes over the NF-κB reporter (Fig. S8 C vs. Fig. 1C), and stimulation of the AP-1 luciferase reporter gene at higher concentrations (Fig. S8 D vs. Fig. 1D). It is worth noting that unlike Psora-4, clofazimine does not significantly cross inhibit Kv1.5, which should make clofazimine less toxic. Although all existing experimental evidence is consistent with Kv1.3 as a molecular target for clofazimine, we cannot rule out other molecular targets for clofazimine, since it has not been possible to take a unbiased approach, such as photoaffinity labeling of whole cell lysates [41], to detect clofazimine binding proteins.

Although we observed excellent correlation between inhibition of Kv1.3 (Fig. 3) and that of IL-2 reporter gene (Fig. 1) as well as that of thapsigargin-induced calcium oscillation in Jurkat T cells (Fig. 2C–D), one discrepancy remains—the calcium influx in only a subpopulation of Jurkat T cells, as measured by the fluorescent dye Fura-2AM, is completely inhibited (Fig. 2A–B). In addition to Kv1.3, T cells express calcium-activated potassium channel that is responsible for setting up the membrane potential to drive calcium influx and that, together with TRPM4 and Kv1.3, is responsible for calcium oscillation in T cells [33]. In Jurkat T cells, this calcium-activated potassium channel is SKCa2 [42]. The varying degrees of sensitivity of individual Jurkat T cells in a given population to clofazimine could be attributed to the different levels of expression of SKCa2 in them.

There are two related but distinct parameters for measuring calcium influx, one being the amount of calcium ions entering T cells via the CRAC channel, which is measured by the Fura-2AM dye (Fig. 2A–B) and the other being calcium oscillation frequency (Fig. 2C–D). It has been shown that both a sufficient amplitude of calcium current and an appropriate oscillation frequency are essential for activating downstream signaling events [30]. In Jurkat T cells, three ion channels other than CRAC have been shown to modulate calcium influx and oscillations, namely TRPM4, Kv1.3 and small-conductance calcium-activated potassium channel, or hSKCa2 [33]. We did observe a strong correlation between the effect of clofazimine on calcium oscillation frequency (Fig. 2C–D) and Kv1.3 inhibition (Fig. 3). Together with previous reports on the important role of calcium oscillation frequency in signaling and cytokine gene expression in T cells, these results offer a coherent and consistent mechanism of action by clofazimine via inhibition of Kv1.3. However, at the same concentration of clofazime that inhibited calcium oscillation frequency in over 80% cells, only 25–50% are affected significantly in the amount of calcium ions getting into cells (Fig. 2A–B). Thus, even among those “non-responsive” cells as judged by the change in net cytosolic calcium concentrations, clofazimine inhibited the calcium oscillation in them (Fig. 2. A–B vs. C–D). The same discrepancy was observed with Psora-4, a well-established Kv1.3 inhibitor (Fig. S8A–B and data not shown). On the other hand, it has been reported that several selective and potent pharmacological inhibitors of KCa channels, but not KV channels, reduce Ca^2+^ entry in Jurkat and in mitogen-activated human T cells [42]. Together, these results strongly suggest that the two potassium channels involved in regulating CRAC current have distinct functions. While it is unclear whether KCa channels regulate both the amount of Ca^2+^ entry and oscillation frequency, it is apparent that Kv1.3 is mainly responsible for enabling oscillation frequency with less influence on the amount of Ca^2+^ entry (Fig. 2). To the best of our knowledge, this is the first time that a causal relationship between Kv1.3 and calcium oscillation frequency has been directly demonstrated experimentally.

As a new member of a growing family of inhibitors for Kv1.3, clofazimine is unique in its chemical structure. The tricyclic phenazine core of clofazimine has not been seen in any other known inhibitors of Kv1.3, thus providing a new pharmacophore for structure/activity relationship studies and the development of new generations of more potent inhibitors. On the other hand, clofazimine does share some similarity with known Kv1.3 inhibitors in certain structural features. For example, the polycyclic core is reminiscent of the tricyclic core of Psora-4 and bicyclic cores of WIN173173. The chlorophenyl substituents on clofazimine can also be found in WIN173173. Whether these gross structural similarities can be translated into similar binding modes of these Kv1.3 inhibitors remains to be determined.

Clofazimine has been used as an anti-leprosy drug since the early 1960s. In addition to leprosy, clofazimine has also been tested and found to be efficacious in several autoimmune diseases including lupus erythematosus [43], [44], Crohn's disease [45], ulcerative colitis [46], and pustular psoriasis [47]. In particular, it was shown to be effective in human graft-versus-host disease [48]. To date, however, the molecular mechanism of its immunomodulatory activity as well as its anti-mycobacterial activity has remained unknown. The surprising connection between clofazimine and the Kv1.3 potassium channel made in this manuscript not only unraveled a fundamental molecular mechanism of action of clofazimine in the human immune system, but also suggested potentially new expanded therapeutic applications of clofazimine. It is fascinating that clofazimine has been used in a recent trial as part of a combined antibiotic regimen in Crohn's disease to test the hypothesis that Mycobacterium avium subspecies paratuberculosis is a possible cause of the disease [49]. Although a suboptimal dose of clofazimine (50 mg/day) was administered along with other drugs, the outcome of the clinical trial may have to be reinterpreted in light of the inhibitory effect of clofazimine on effector memory T cells uncovered in the present study [50]. In addition to T cell-mediated graft rejection, Kv1.3 has been shown to play a uniquely important role in the proliferation and survival of effector memory T cells which have been implicated in the pathogenesis of a number of autoimmune diseases. Thus, clofazimine is likely to be effective on a number of autoimmune disorders through the same molecular mechanism.

Although clofazimine does have a number of side effects including GI and skin discoloration [51]–[53], it is well tolerated and relatively safe [54]. In particular, it lacks the nephrotoxicity and neurotoxicity associated with both CsA and FK506 [55]. As such, clofazimine has great potential in the treatment of autoimmune diseases such as multiple sclerosis, type 1 diabetes and psoriasis as well as organ transplantation. The fact that clofazimine, unlike other known inhibitors of Kv1.3, has been used in humans for decades may allow for its accelerated clinical evaluation and eventual introduction as new treatments for different autoimmune and other pertinent diseases.

## Materials and Methods

### Cell culture and IL2-Luciferase reporter cell line

Jurkat E6.1 (ATCC TIB152) T cells were maintained in RPMI medium 1640 (Invitrogen) supplemented with 10% FBS, 2 mM-L-glutamine, penicillin (50 µg/ml) and streptomycin (50 µg/ml). HFF-1 (ATCC SCRC1041) fibroblast was maintained in low glucose DMEM (Invitrogen) supplemented with 10% FBS, 2 mM-L-glutamine, penicillin (50 µg/ml) and streptomycin (50 µg/ml). Jurkat E6.1 cells were transfected with linearized pIL2-Luc-Neo and linearized pMEP4 by electroporation, followed by selection for resistance to 400 µg/ml hygromycin. The stably transfected IL-2 reporter cell line, Jurkat/IL-2Luc, was maintained in hygromycin, which was omitted from medium for the screening and other assays.

### The Hopkins Drug Library (HDL)

An early version of the library has been described [24]. Clofazimine was purchased from Sigma.

### Screening assay

Jurkat/IL-2Luc cells were seeded in 96 well plates (Nunc 136102) at 2×10^5^ (180 µL) per well. Cells were incubated with 10 µM (final concentration) drugs from the library for 1 h before they were stimulated with 1 µM ionomycin and 40 nM phorbol myristate acetate (PMA) for an additional 16 h. Cells were pelleted by centrifugation. Upon removal of the culture medium, lysis/assay buffer was added into each well. The luciferase activity was determined as per manufacture's instructions.

### Calcein incorporation assay

HFF-1 (human forebrain fibroblast) cells (2×10^3^ in 190 µl) were seeded into 96-well plates and were incubated with drugs from the library for 4 days. Cells were washed with PBS twice before they were treated with 1 µM (final concentration) Calcein-AM (invitrogen C1430) for 4 h. Plates were directly counted by a fluorescent plate reader.

### Detection of IL-2 secreted from T cells

Jurkat E6.1 T cells (1×10^5^ in 180 µl) were seeded into 96-well plates. Cells in each well were treated with different concentrations of clofazimine for 1 h before 1 µM ionomycin and 40 nM PMA were added. The incubation was continued for 2 days. The plates were centrifuged at 1,200×g for 5 min, and the supernatant from each well was collected followed by ELISA detection of IL-2. The primary antibody, biotinylated secondary antibody and HRP conjugated avidin were purchase from BD/Pharmingen.

### Dephosphorylation of endogenous NFATc2

Jurkat T cells were pretreated with indicated compounds for 1 h before 3 µM (final concentration) ionomycin was added. Cells were harvested after 30 min and lysed in a lysis buffer [40 mM Tris (pH 7.8), 1% NP-40, 10 mM EDTA, 60 mM Na_3_P_4_O_7_ and common protease inhibitors] by sonication. NFATc2 was resolved by 8% SDS-PAGE followed by Western blot analysis using anti-NFAT antibodies (Santa-cruz Sc-7296, 1∶100 dilution).

### Calcium imaging assay

Jurkat cells were attached to L-lysine-coated glass dishes (MatTek). Cells were loaded with 1 µM fura-2-AM (Invitrogen F-1201) for 45 min before they were washed sequentially with growth medium and Ca^2+^-free HBSS (Hank's buffer with 2 g/L glucose and 20 mM HEPES, pH 7.4). Fluorescence excitation wavelengths were set at 350 and 380 nm, respectively, on a Zeiss Axiovert 200M microscope while emission wavelength was set at 510 nm. Changes in intracellular calcium concentrations were determined by fluorescence intensity ratio (F_350_/F_380_).

### Measurement of calcium oscillation period

Calcium oscillation was stimulated by 10 nM TG and 5 µM clofazimine was added 30 min after initiation of oscillation by TG. The average oscillation periods before and after drug addition were determined by total time divided by peak numbers. Changes in average oscillation period larger than 10 seconds were considered significant while those smaller than 10 seconds were ignored for statistical analysis.

### Patch-clamp experiments

Patch pipettes, pulled from glass capillaries (inner diameter 1.5 mm, Kimble products) with a horizontal puller (Sutter instruments, Modell P-97), were fire-polished, and had resistances between 2 and 4 MOhm. Patch-clamp experiments were performed in the tight-seal whole-cell configuration at room temperature (22–24°C). High-resolution current recordings were acquired by a computer-based patch-clamp amplifier system (EPC-9, HEKA). Immediately following establishment of the whole-cell configuration, every two seconds voltage ramps of 50 ms duration spanning the voltage range of −100 to +100 mV for Jurkat T cells and −150 to +150 mV for HEK293 were delivered from a holding potential (V_h_) of −70 mV for Jurkats and 0 mV for HEK293 cells over a period of 300–600 s. Heterologously expressed potassium currents (Kv1.1, Kv1.2, Kv1.3, Kv1.5 and Kv3.1) were acquired using a voltage ramp from −100 mV to +100 mV over 500 ms and at 2 s (Kv3.1 & Kv1.2) or 5 s intervals from a V_h_ of −80 mV. All voltages were corrected for a liquid junction potential of 10 mV. Currents were filtered at 2.9 kHz and digitized at 100 ms intervals. Capacitive currents and series resistance were determined and corrected before each voltage ramp using the automatic capacitance compensation of the EPC-9. Kv1.3 currents were normalized to the point of application of 5 µM clofazimine. Kv1.3 currents in Jurkat T cells were measured at +80 mV. Heterologously expressed Kv1.1, Kv1.2, Kv1.3, Kv1.5 and Kv3.1 were assessed at +80 mV except in Fig. S5, where currents were additionally measured at the more physiological voltage of 0 mV. CRAC currents in HEK293 cells were assessed at −80 mV. Standard external solution contained (in mM): 140 NaCl, 1 CaCl_2_, 2 MgCl_2_, 2.8 KCl, 10 HEPES-NaOH, with pH at 7.2 and osmolarity 300–320 mosm. The solution contained 10 mM CaCl_2_ in the case of CRAC measurements. Internal standard solution contained (in mM): 120 K-glutamate, 1 MgCl_2_, 8 NaCl, 10 HEPES-KOH, 10 K-BAPTA, with pH 7.2 and osmolarity 290–310 mosm. The internal solution was supplemented with 20 µM IP_3_ for CRAC measurements. Substance application was performed on individual cells using a wide-mouth glass pipette connected to a pneumatic pressure device.

### Immunofluorescence

Jurkat T cells treated with clofazimine were centrifuged onto L-lysine-coated coverslips. Cells were fixed with 4% *p*-formaldehyde for 15 min. The cells were washed in PBS, permeabilized by −20°C methanol and blocked with 10% donkey serum in PBS. The fixed cells were then incubated with Kv1.3 primary antibody (sc-17239 at 1∶100 dilution) for 1 h. Cells were washed in PBS (3×5 min) and incubated with donkey anti-goat Cy5 antibodies for an additional 1 h. Cells were then washed three times in PBS, mounted, and photographed. Mounting was performed using Vectashield mounting medium (Vector Laboratories) and images were captured using either a Zeiss LSM510 confocal microscope. Merged images were compiled using LSM5 Image Examiner or Adobe Photoshop CS.

### shRNA Lentivirus Production

The targeting sequence of sh Kv1.3-4 is 5′-GCCACCTTCTCGCGAAACAT-3′. Recombinant lentiviruses were generated using a three-plasmid system described previously [56]. Virus was harvested and concentrated (1∶50) 2 days after transfection. Jurkat T cells were transduced by concentrated virus at a ratio of 5×10^6^ cells/150 µl virus. Cells were cultured for two days before they were harvested for Western blot analysis and other experiments.

### Clofazimine gel-shift assay

The lower resolving gel was cast with 6% acrylamide, 375 mM Tris (pH 8.5), 0.1% APS and 0.04% TEMED. The upper stacking gel was cast with 5% acrylamide, 125 mM Tris (pH 6.8), 0.1% APS and 0.1% TEMED. Clofazimine was incubated with or without indicated protein in a buffer (20 mM Tris (pH 7.4), 200 mM KCl, 0.1% phosphatidylcholine, 1 mM iodoacetamide and 2% CHAPS) for 30 minutes before aliquots of 5×loading buffer (50% glycerol, 1M Tris (pH 8.5)) was added. The running buffer was made from 14.4 g/l glycine and pH was adjusted by Tris to 8.5. Electrophoresis was carried out overnight at 4°C with stable current of 11 mA. The proteins and clofazimine were transferring onto a nitrocellulose membrane in regular transfer buffer with 0.1% SDS. Once on the membrane, clofazimine can be visualized directly as a light yellow band. The membrane was then subject to staining with anti-Kv1.3 antibodies to locate the protein band.

### Western blot analysis of Kv1.3

Jurkat T cells were washed once in PBS and lysed by RIPA buffer with 5 mM PMSF, 10 µg/ml each of pepstatin, leupeptin and aprotinin. The protein concentrations were determined by Bradford assay before the lysate was boiled with loading buffer and resolved by SDS-PAGE. Proteins were transferred onto a nitrocellulose membrane under 100 volts for 1 h. The membrane was blotted with anti-kv1.3 antibody (sc-17241, Santa Cruz) with a dilution of 1∶200 in 5% milk followed by wash and incubation with HRP-conjugated donkey anti-goat IgG (sc-2020). The membrane was developed using an ECL kit (Pierce 34078).

### Human mixed lymphocyte reaction (MLR)

The human MLR was established by coculturing normal human PBMN responder lymphocytes (0.5×10^6^) with an equal number of miomycin C-treated stimulator cells in RPMI1640 complete medium. The cells were incubated with varying doses of clofazimine at 37°C in a humidified atmosphere of 5% CO_2_ for 4 days. Then 1 µCi of [^3^H]-thymidine was added into culture and incubation was continued for 6 h. The cells were harvested and [^3^H]-thymidine incorporated into cells was measured in a liquid scintillation counter.

### Mouse model of human skin transplantation

The experimental procedure is similar to that described previously [37]. Clofazimine suspension in olive oil was given p.o. at 50 mg/kg/day in a total volume of 0.2 ml per administration.

For additional experimental procedures, see Materials and Methods S1

## Supporting Information

Figure S1 Effects of clofazimine on AP-1 luciferase reporter gene and the nuclear translocation of NFAT in response to ionomycin treatment. (A) Dose-dependent enhancement of the AP-1 luciferase reporter gene by CsA (n = 6). (B) Clofazimine inhibits EGFP-NFATc3 nuclear translocation in Jurkat T cells stimulated by 1 µM ionomycin. Images were taken 30 minutes after addition of ionomycin. (C) Dose-dependent inhibition of ionomycin-stimulated NFAT nuclear translocation by clofazimine (n = 3).
(0.18 MB TIF)

Figure S2 Clofazimine does not affect the enzymatic activity of calcineurin in vitro. (A) Clofazimine does not inhibit the protein phosphatase activity of calcineurin in vitro. 20 mM p-nitrophenylphosphate was incubated with purified recombinant calcineurin A/B and calmodulin in the presence of 1 mM calcium or 5 mM EGTA at 30°C. The progress of the reaction was followed by absorbance at 410 nm every 0.5 second. Representative curves of three different experiments. (B) Clofazimine does not inhibit NFATc2 dephosphorylation by calcineurin in vitro. NFATc2 was immuno-precipitated from Jurkat lysate and incubated with recombinant calcineurin A/B and calmodulin for 30 min at room temperature in the presence of 1 mM Ca2+ or 5 mM EGTA. The reaction mixtures were subjected to SDS-PAGE, followed by Western blot using anti-NFAT antibodies. (C) Clofazimine does not interfere with calcineurin-NFATc2 interaction. GST-NFATc2 (1–415) was purified by glutathione-sepharose beads and incubated with Jurkat cell lysate. The pull-down products were resolved by SDS-PAGE and detected by α-calcineurin antibody. (D) Clofazimine does not affect binding calcineurinA (1–400, H160N) and NFATc2 (1–415) in Jurkat T cells in a mammalian two-hybrid assay. But it inhibits the calcium-dependent enhancement of the calcineurin-NFATc2 interaction. (n = 6)
(0.35 MB TIF)

Figure S3 Clofazimine alters calcium oscillation patterns in Jurkat T cells without affecting reconstituted ICRAC in HEK293 cells. (A, B) Time-dependent increase in the population of cells that are sensitive to clofazimine. Jurkat T cells were incubated with clofazimine for varied lengths of time before 1 µM TG was added. Images were taken 30 min after 2 mM calcium was added. (C) Average CRAC current densities at −80 mV induced by IP3 (20 µM) in stable STIM1 expressing HEK293 cells transiently overexpressing CRACM1, CRACM2 and CRACM3. Holding potential was at 0 mV. The bar indicates the time for clofazimine application.
(0.56 MB TIF)

Figure S4 Effect of 10 µM clofazimine on heterologous Kv1.1, Kv1.2 Kv1.5 and Kv3.1. (A) Average time course of mouse Kv1.1 currents stably expressed in L929 cells. Control cells (closed circles, n = 5, no application) and cells superfused with 10 µM clofazimine added to the standard extracellular solution (open circles, n = 6) as indicated by the black bar. Voltage protocol, solutions and analysis as outlined in Fig. 3D. (B) Current-voltage relationship (I/V) of a representative cell expressing mouse Kv1.1 with control I/V (black) extracted at 40 s after whole-cell establishment and the I/V for clofazimine extracted at the end of application (red, 160 s). (C) Average time course of heterologous rat Kv1.2 expressed in B82 cells. Control cells (closed circles, n = 5, no application) and cells superfused with 10 µM clofazimine (open circles, n = 5, black bar indicates application time) are shown. Acquisition and analysis as in (A). (D) I/V of a representative cell expressing rat Kv1.2 with control I/V (black) extracted at 40 s after whole-cell establishment and the I/V for clofazimine extracted at the end of application (red, 160 s). (E) Average time course of heterologous human Kv1.5 expressed in MEL cells. Control cells (closed circles, n = 5, no application) and cells superfused with 10 µM clofazimine (open circles, n = 5, black bar indicates application time) are shown. Acquisition and analysis as in (A). (F) I/V of a representative cell expressing human Kv1.5 with control I/V (black) extracted at 40 s after whole-cell establishment and the I/V for clofazimine extracted at the end of application (red, 160 s). (G) Average time course of heterologous mouse Kv3.1 expressed in L929 cells. Control cells (closed circles, n = 5, no application) and cells superfused with 10 µM clofazimine (open circles, n = 5, black bar indicates application time) are shown. Acquisition and analysis as in (A). (H) I/V of a representative cell expressing mouse Kv3.1 with control I/V (black) extracted at 40 s after whole-cell establishment and the I/V for clofazimine extracted at the end of application (red, 160 s).
(0.41 MB TIF)

Figure S5 Inhibition of Kv channels by 10 µM clofazimine assessed at 0 mV (red bars) or +80 mV (black bars). Same cells as in Fig. 3D and Fig. S4 were used. Note the increased inhibitory effect at 0 mV for all Kv channels displayed except Kv1.3.
(0.19 MB TIF)

Figure S6 Knockdown of Kv1.3 did not affect the sensitivity of the IL-2 luciferase reporter to CsA. The IC50 for control EGFP-siRNA lentiviral transduced Jurkat T cells was 2.5±0.6 nM, and that for Kv1.3 lentiviral 4 transduced T cells was 2.6±0.7 nM (n = 6).
(0.00 MB TIF)

Figure S7 Effects of clofazimine on mouse TCR-mediated IL-2 production and mixed lymphocyte reaction in murine T cells. (A) Dose response of IL-2 production from anti-CD3/anti-CD28-stimulated mouse primary T cells to different concentrations of clofazimine (n = 3). (B) Biphasic effects of clofazimine on mouse mixed lymphocyte reaction (n = 3).
(0.00 MB TIF)

Figure S8 Effects of Psora-4 on calcium influx and the activation of different reporter genes in Jurkat T cells. (A) Calcium influx in Jurkat T cells was inhibited by 10 µM psora-4 in a heterogeneous fashion. Psora-4 was added 5 minutes before stimulation with 1 µM TG. Representative images were taken 30 minutes after 2 mM calcium was added. The color gradient represents fura-2 at 350 nm/380 nm excitation ratio. (B) Quantitation of 350 nm/380 nm ratios for calcium imaging results shown in (A). Jurkat T cells can be divided into psora-4 responsive (R) and psora-4 non-responsive (NR) groups. (C) Psora-4 inhibits NFAT pathway in Jurkat T cells. The IC50s of psora-4 for IL-2, NFAT reporters are 9.3±1.7 µM and 29.6±8.2 µM, respectively. And the IC50 of clofazimine for NF-κB luciferase reporter assay is over 1 mM (n = 6 each). All the reporters were stimulated with PMA/ionomycin. (D) Psora-4 significantly enhances AP-1 luciferase reporter at high concentrations (>50 µM, n = 6), similar to clofazimine.
(0.77 MB TIF)

Materials and Methods S1 Supporting Materials and Methods
(0.10 MB DOC)
