# Supplementary figures and images for: Clofazimine Inhibits Human Kv1.3 Potassium Channel by Perturbing Calcium Oscillation in T Lymphocytes
Source: PLoS One. 2008 Dec 23;3(12):e4009. doi: 10.1371/journal.pone.0004009 (PMC2602975; doi:10.1371/journal.pone.0004009)

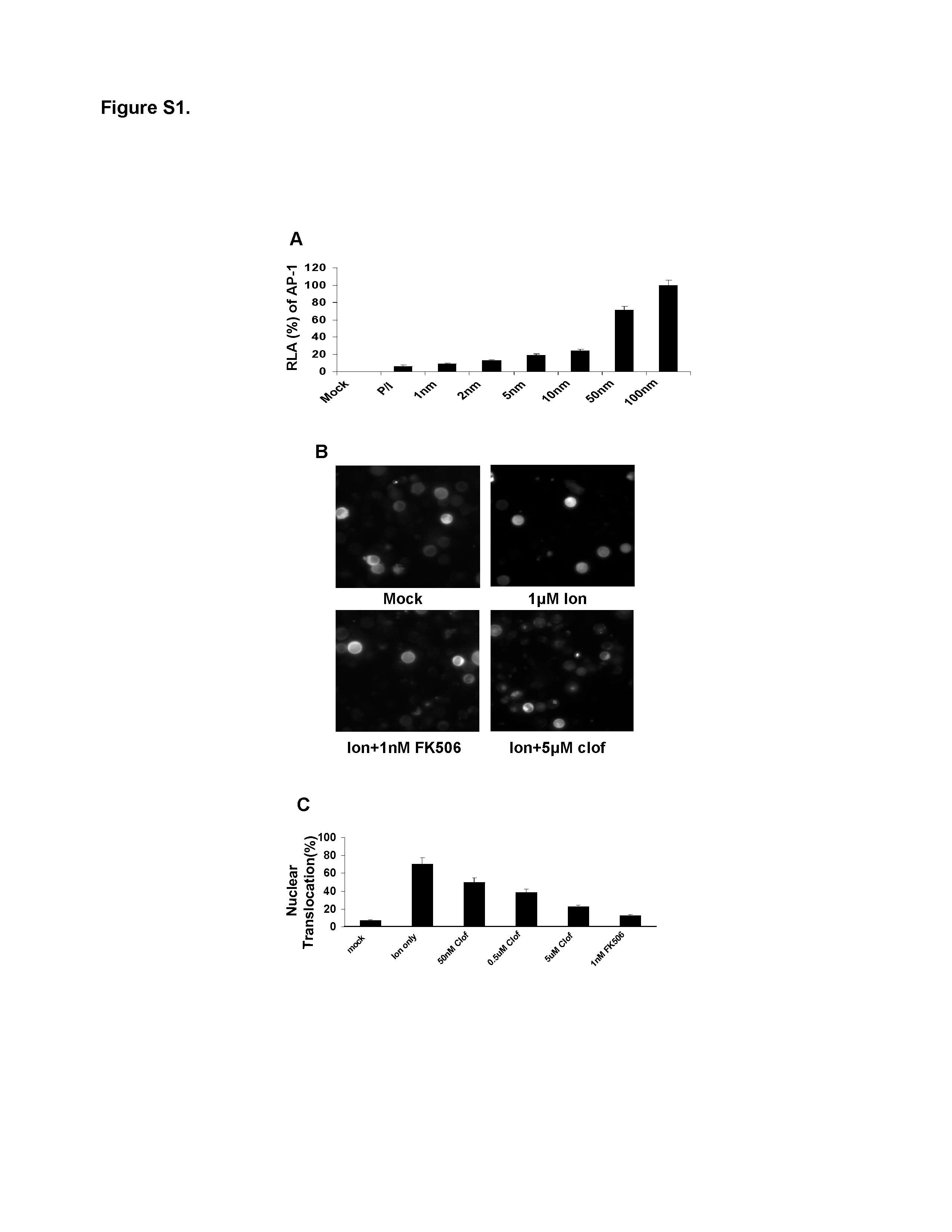

Supplement: Figure S1 — Effects of clofazimine on AP-1 luciferase reporter gene and the nuclear translocation of NFAT in response to ionomycin treatment. (A) Dose-dependent enhancement of the AP-1 luciferase reporter gene by CsA (n = 6). (B) Clofazimine inhibits EGFP-NFATc3 nuclear translocation in Jurkat T cells stimulated by 1 µM ionomycin. Images were taken 30 minutes after addition of ionomycin. (C) Dose-dependent inhibition of ionomycin-stimulated NFAT nuclear translocation by clofazimine (n = 3). (0.18 MB TIF) [file pone.0004009.s001.tif]

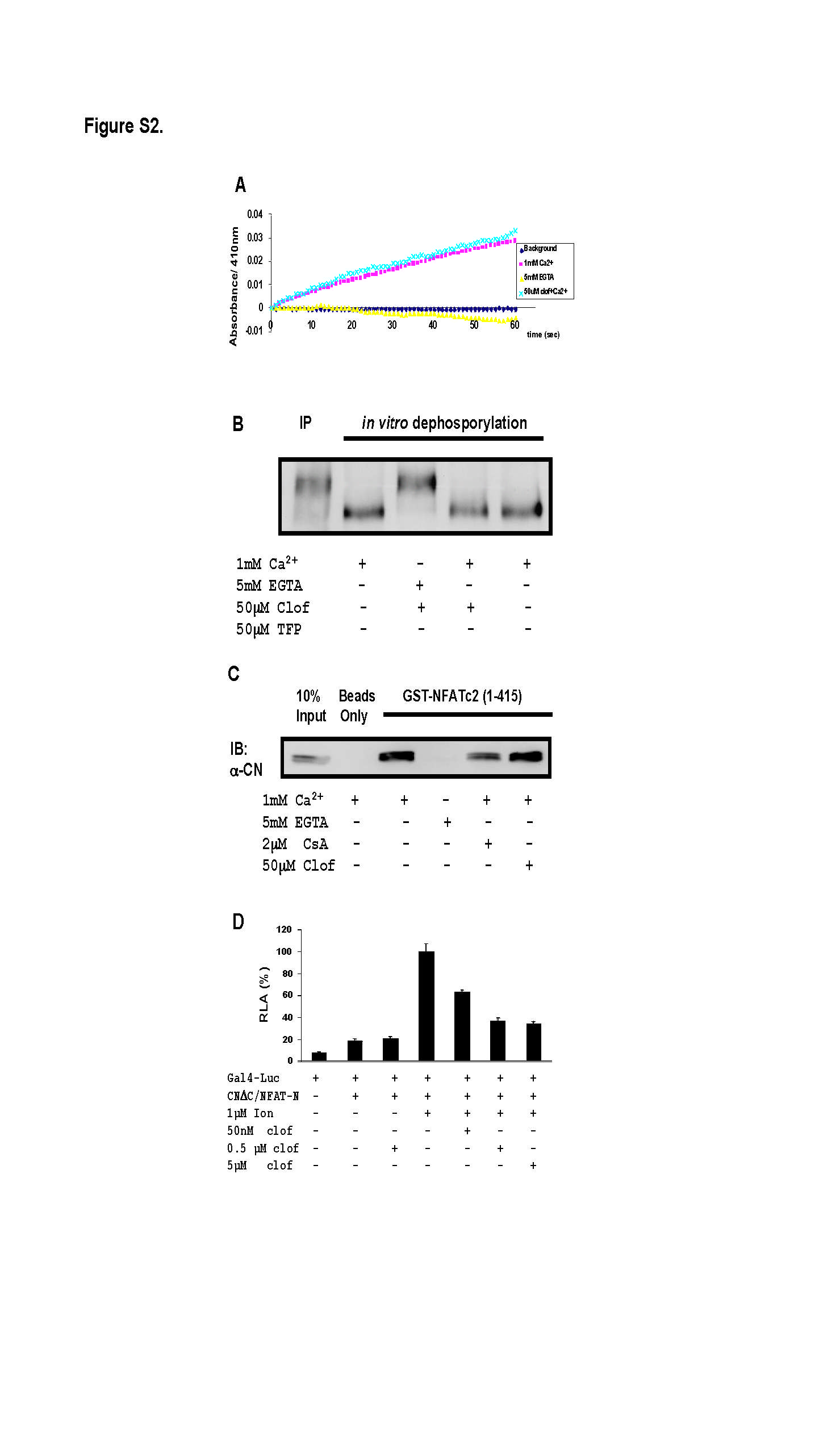

Supplement: Figure S2 — Clofazimine does not affect the enzymatic activity of calcineurin in vitro. (A) Clofazimine does not inhibit the protein phosphatase activity of calcineurin in vitro. 20 mM p-nitrophenylphosphate was incubated with purified recombinant calcineurin A/B and calmodulin in the presence of 1 mM calcium or 5 mM EGTA at 30°C. The progress of the reaction was followed by absorbance at 410 nm every 0.5 second. Representative curves of three different experiments. (B) Clofazimine does not inhibit NFATc2 dephosphorylation by calcineurin in vitro. NFATc2 was immuno-precipitated from Jurkat lysate and incubated with recombinant calcineurin A/B and calmodulin for 30 min at room temperature in the presence of 1 mM Ca2+ or 5 mM EGTA. The reaction mixtures were subjected to SDS-PAGE, followed by Western blot using anti-NFAT antibodies. (C) Clofazimine does not interfere with calcineurin-NFATc2 interaction. GST-NFATc2 (1–415) was purified by glutathione-sepharose beads and incubated with Jurkat cell lysate. The pull-down products were resolved by SDS-PAGE and detected by α-calcineurin antibody. (D) Clofazimine does not affect binding calcineurinA (1–400, H160N) and NFATc2 (1–415) in Jurkat T cells in a mammalian two-hybrid assay. But it inhibits the calcium-dependent enhancement of the calcineurin-NFATc2 interaction. (n = 6) (0.35 MB TIF) [file pone.0004009.s002.tif]

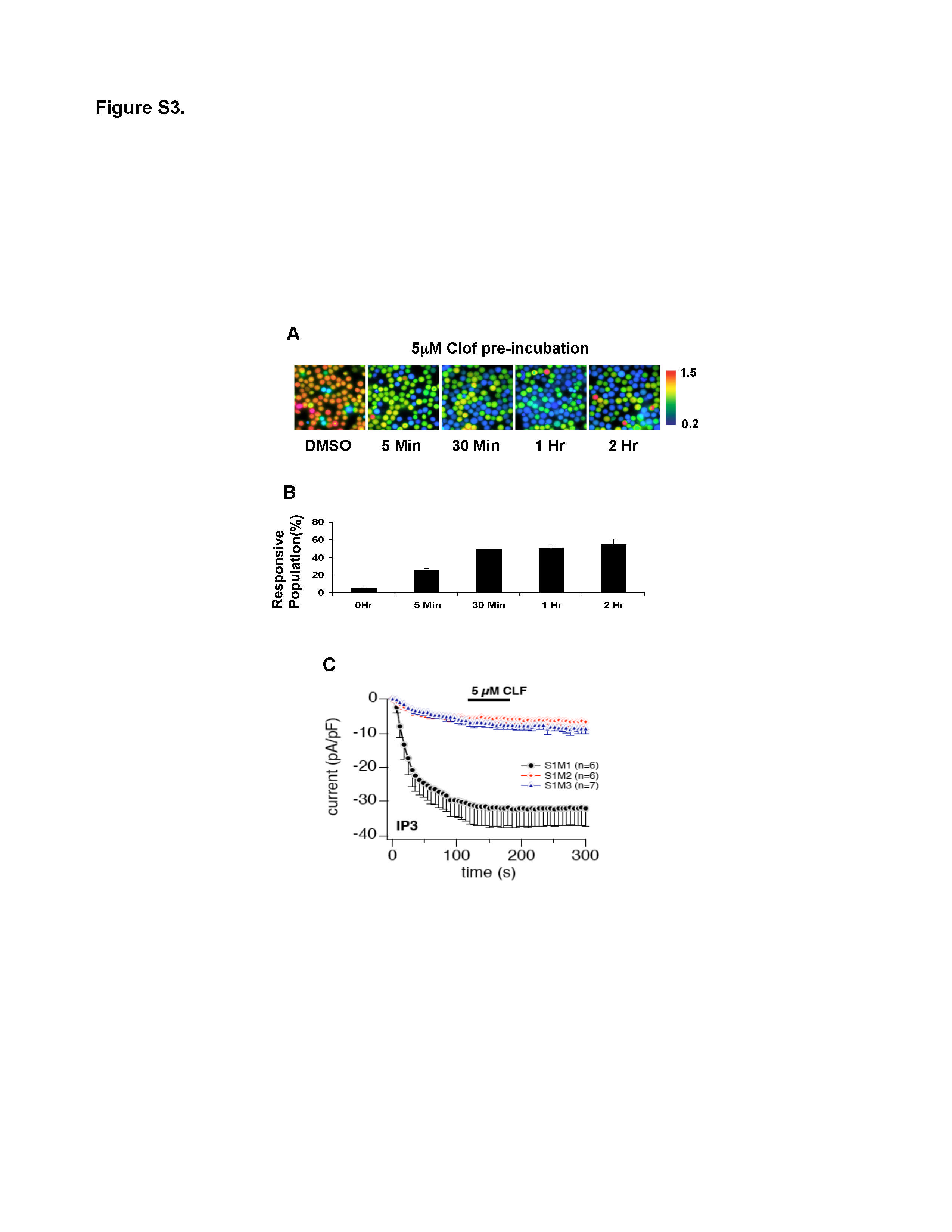

Supplement: Figure S3 — Clofazimine alters calcium oscillation patterns in Jurkat T cells without affecting reconstituted ICRAC in HEK293 cells. (A, B) Time-dependent increase in the population of cells that are sensitive to clofazimine. Jurkat T cells were incubated with clofazimine for varied lengths of time before 1 µM TG was added. Images were taken 30 min after 2 mM calcium was added. (C) Average CRAC current densities at −80 mV induced by IP3 (20 µM) in stable STIM1 expressing HEK293 cells transiently overexpressing CRACM1, CRACM2 and CRACM3. Holding potential was at 0 mV. The bar indicates the time for clofazimine application. (0.56 MB TIF) [file pone.0004009.s003.tif]

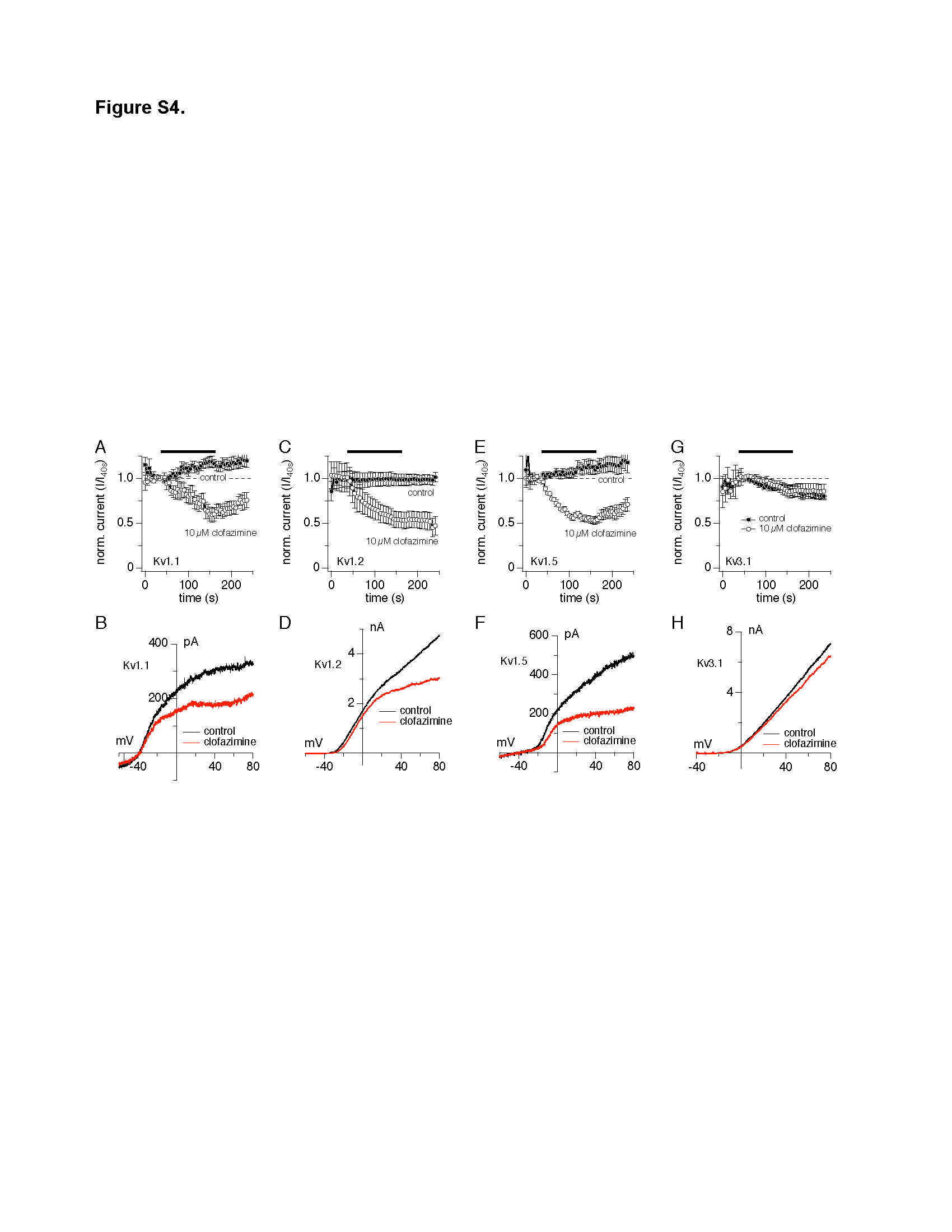

Supplement: Figure S4 — Effect of 10 µM clofazimine on heterologous Kv1.1, Kv1.2 Kv1.5 and Kv3.1. (A) Average time course of mouse Kv1.1 currents stably expressed in L929 cells. Control cells (closed circles, n = 5, no application) and cells superfused with 10 µM clofazimine added to the standard extracellular solution (open circles, n = 6) as indicated by the black bar. Voltage protocol, solutions and analysis as outlined in Fig. 3D. (B) Current-voltage relationship (I/V) of a representative cell expressing mouse Kv1.1 with control I/V (black) extracted at 40 s after whole-cell establishment and the I/V for clofazimine extracted at the end of application (red, 160 s). (C) Average time course of heterologous rat Kv1.2 expressed in B82 cells. Control cells (closed circles, n = 5, no application) and cells superfused with 10 µM clofazimine (open circles, n = 5, black bar indicates application time) are shown. Acquisition and analysis as in (A). (D) I/V of a representative cell expressing rat Kv1.2 with control I/V (black) extracted at 40 s after whole-cell establishment and the I/V for clofazimine extracted at the end of application (red, 160 s). (E) Average time course of heterologous human Kv1.5 expressed in MEL cells. Control cells (closed circles, n = 5, no application) and cells superfused with 10 µM clofazimine (open circles, n = 5, black bar indicates application time) are shown. Acquisition and analysis as in (A). (F) I/V of a representative cell expressing human Kv1.5 with control I/V (black) extracted at 40 s after whole-cell establishment and the I/V for clofazimine extracted at the end of application (red, 160 s). (G) Average time course of heterologous mouse Kv3.1 expressed in L929 cells. Control cells (closed circles, n = 5, no application) and cells superfused with 10 µM clofazimine (open circles, n = 5, black bar indicates application time) are shown. Acquisition and analysis as in (A). (H) I/V of a representative cell expressing mouse Kv3.1 with control I/V (black) extracted [file pone.0004009.s004.tif]

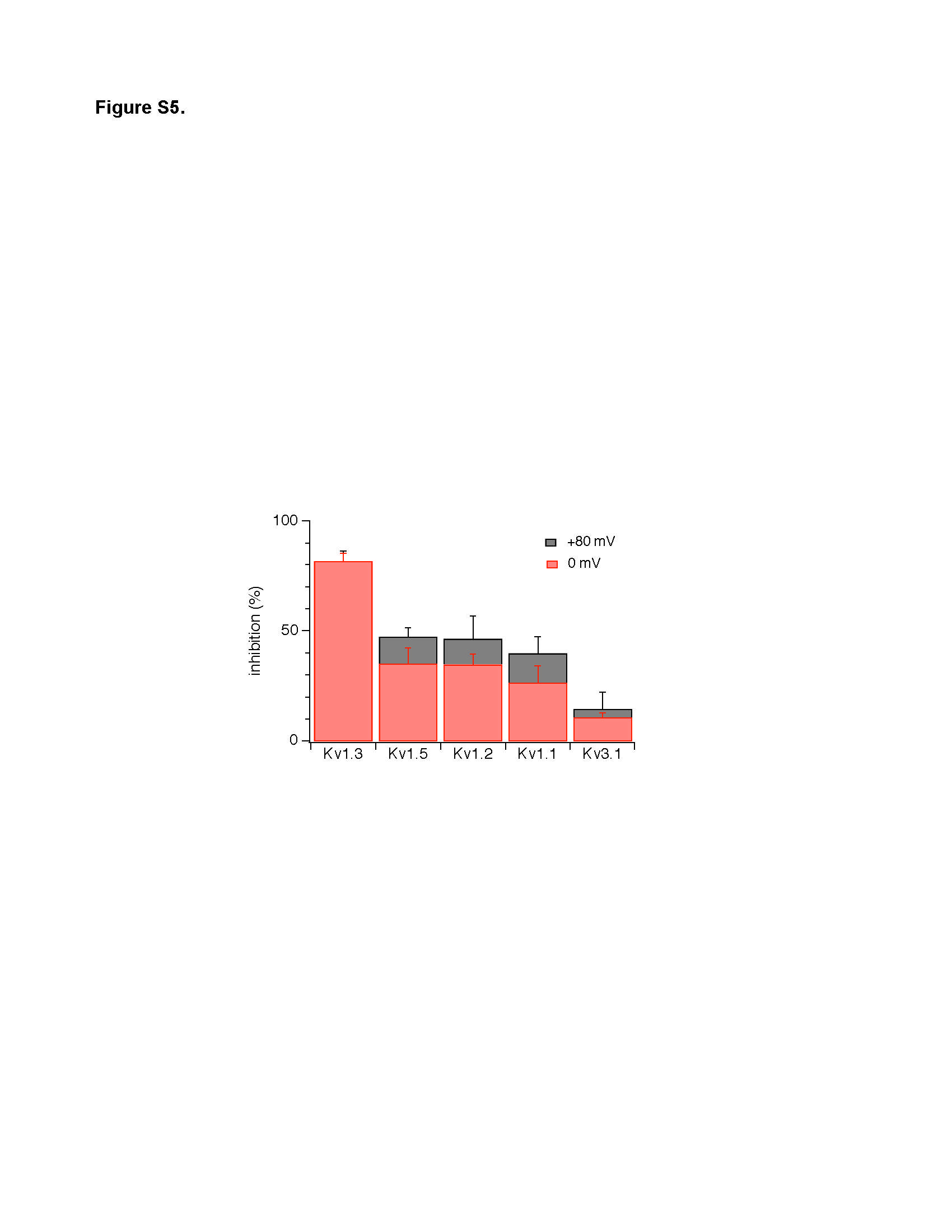

Supplement: Figure S5 — Inhibition of Kv channels by 10 µM clofazimine assessed at 0 mV (red bars) or +80 mV (black bars). Same cells as in Fig. 3D and Fig. S4 were used. Note the increased inhibitory effect at 0 mV for all Kv channels displayed except Kv1.3. (0.19 MB TIF) [file pone.0004009.s005.tif]

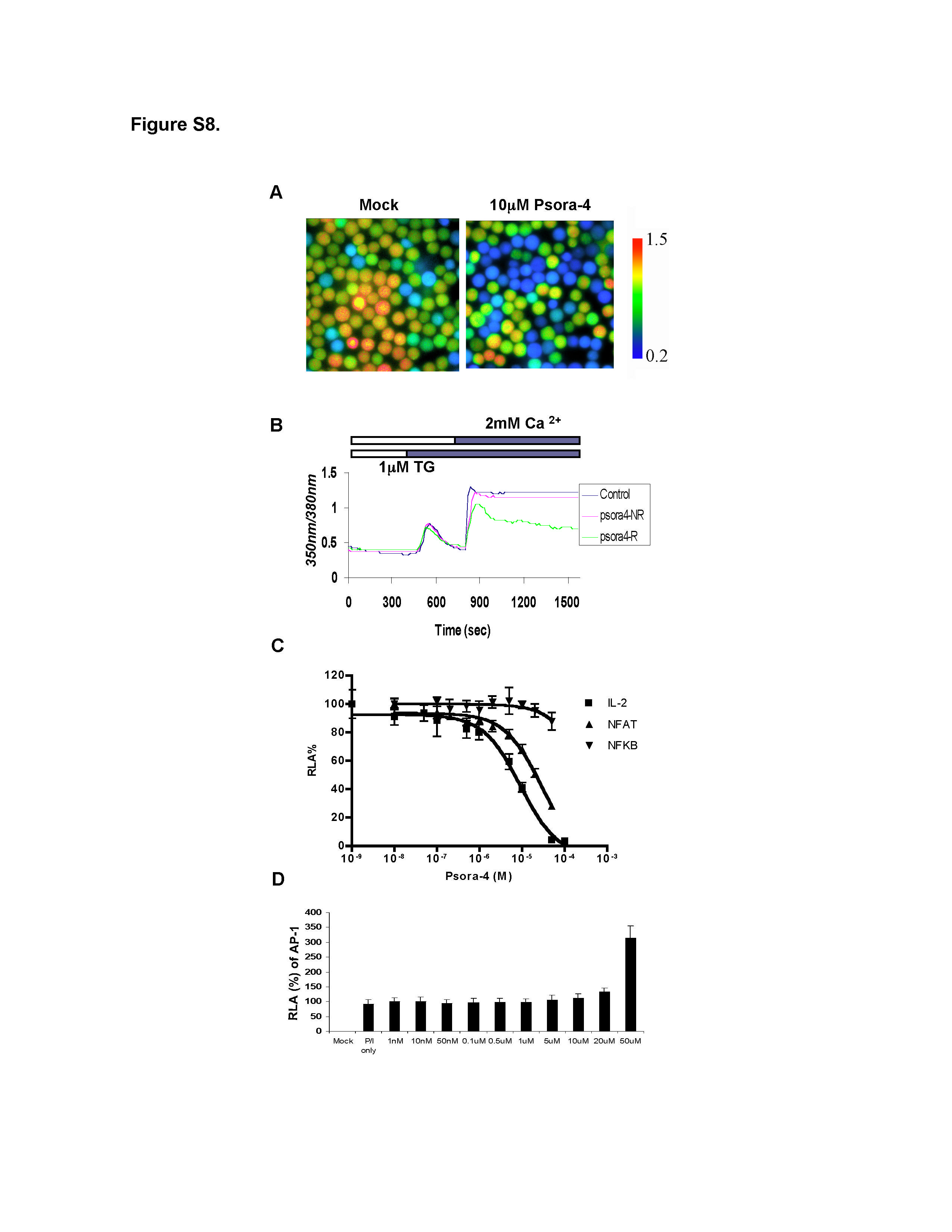

Supplement: Figure S8 — Effects of Psora-4 on calcium influx and the activation of different reporter genes in Jurkat T cells. (A) Calcium influx in Jurkat T cells was inhibited by 10 µM psora-4 in a heterogeneous fashion. Psora-4 was added 5 minutes before stimulation with 1 µM TG. Representative images were taken 30 minutes after 2 mM calcium was added. The color gradient represents fura-2 at 350 nm/380 nm excitation ratio. (B) Quantitation of 350 nm/380 nm ratios for calcium imaging results shown in (A). Jurkat T cells can be divided into psora-4 responsive (R) and psora-4 non-responsive (NR) groups. (C) Psora-4 inhibits NFAT pathway in Jurkat T cells. The IC50s of psora-4 for IL-2, NFAT reporters are 9.3±1.7 µM and 29.6±8.2 µM, respectively. And the IC50 of clofazimine for NF-κB luciferase reporter assay is over 1 mM (n = 6 each). All the reporters were stimulated with PMA/ionomycin. (D) Psora-4 significantly enhances AP-1 luciferase reporter at high concentrations (>50 µM, n = 6), similar to clofazimine. (0.77 MB TIF) [file pone.0004009.s008.tif]
