# Supplementary material for: Clofazimine Inhibits Human Kv1.3 Potassium Channel by Perturbing Calcium Oscillation in T Lymphocytes
Source: PLoS One. 2008 Dec 23;3(12):e4009. doi: 10.1371/journal.pone.0004009 (PMC2602975; doi:10.1371/journal.pone.0004009)
Supplement: Materials and Methods S1 — Supporting Materials and Methods (0.10 MB DOC) [file pone.0004009.s009.doc]

Supplemental Materials and Methods

Nuclear translocation of GFP-NFATc3 in Jurkat cells

GFP-NFATc3 plasmid was a gift from Dr. Frank Mckeon (Harvard Medical School). Jurkat T cells transiently transfected with GFP-NFATc3 were pretreated with indicated compounds for 1 h before 1 μM (final concentration) ionomycin was added. The NFAT nuclear translocation was recorded under a fluorescent microscope. Experiments were repeated 3 times with at least 100 cells counted each time.

In vitro calcineurin enzymatic assay

Recombinant human calcineurin A β2 and calcineurin B were co-expressed and purified as described previously (Mondragon A. et. al. Biochemistry. 1997, 36:4934.). Either 20 mM p-nitrophenylphosphate (sigma PS613) or endogenous NFATc2 immunoprecipitated (Santa-cruz, Sc-7296) from Jurkat cell lysate was incubated with 0.1 μM calcineurin A/B and 1 μM calmodulin in the presence of 1 mM calcium or 5 mM EGTA at 30 °C for varying duration of time. The color change due to hydrolysis of p-nitrophenylphosphate by calcineurin was monitored by an absorbance at 410 nm. The dephosphorylation of NFATc2, which causes a shift in gel mobility, was detected by SDS-PAGE and followed by Western Blot using anti-NFAT antibodies.

Calcineurin-NFAT binding assay

Recombinant GST-NFATc2 (1-415) was expressed in bacteria and purified by glutathione-Sepharose beads. The sepharose beads were incubated with Jurkat cell lysate

for 2 h before 3 washes. The pull-down products were resolved by SDS-PAGE and detected by α-calcineurin antibody (Abcam, ab55869)

Mammalian 2-hybrid assay

pM conjugated calcinerin A (1-400, H160N) and pVP16 conjugated NFATc2 (1-415) mammalian 2-hybrid system were described previously (Pan F. et. al. Feedback inhibition of calcineurin and Ras by a dual inhibitory protein Carabin. Nature. 2007, 445:433.). The Gal4 promoter driven luciferase reporter vector, pM-calcineurin A vector and pVP-NFAT vector were co-transfected into Jurkat T cells. The transiently-transfected Jurkat T cells were pre-treated with clofazimine for 1 hour before ionomycin stimulation. The luciferase signal was detected by a luminescent reader.
